# The occurrence and zoonotic potential of *Cryptosporidium* species in freshwater biota

**DOI:** 10.1186/s13071-023-05827-9

**Published:** 2023-06-21

**Authors:** Laura Hayes, Guy Robinson, Rachel M. Chalmers, Steve J. Ormerod, Anna Paziewska-Harris, Elizabeth A. Chadwick, Isabelle Durance, Jo Cable

**Affiliations:** 1 School of Biosciences, Cardiff University, Cardiff, CF10 3AX UK; 2 Cryptosporidium Reference Unit, Public Health Wales Microbiology, Singleton Hospital, Swansea, SA2 8QA UK; 3 Swansea Medical School, Swansea University, Swansea, SA2 8QA UK; 4 Lukasiewicz Research Network, PORT Polish Centre for Technology Development, Stablowicka 147, 54-066 Wroclaw, Poland

**Keywords:** Cryptosporidiosis, One health, Zoonoses, Fish vectors, *Cryptosporidium hominis*

## Abstract

**Background:**

Protozoan pathogens from the genus *Cryptosporidium* cause the diarrhoeal disease cryptosporidiosis in humans and animals globally. Freshwater biota could act as potential reservoirs or zoonotic sources of *Cryptosporidium* infections for livestock and people, but *Cryptosporidium* occurrence in aquatic biota is largely unexplored. The aim of this study was to investigate the occurrence of *Cryptosporidium* in a range of freshwater organisms in upland rivers across England and Wales.

**Methods:**

Fish were sampled by electrofishing, invertebrate larvae by kick sampling and the otter *Lutra lutra* and mink *Mustela vison* through faecal samples collected opportunistically as part of a nation-wide study. PCR targeting the small subunit ribosomal RNA gene was used to detect *Cryptosporidium* species.

**Results:**

*Cryptosporidium* occurred in just 0.8% of all the samples and in none of 73 samples from nine invertebrate genera. *Cryptosporidium* was detected in two of 2/74 fish samples (2.7%), both salmonids, and in 2/92 otter faecal samples (2.17%), but there were no positive samples in mink (0/24) or the bullhead *Cottus gobio* (0/16).

**Conclusions:**

Low detection rate of human-infective *Cryptosporidium* species in aquatic fauna indicates they may present a low risk of contamination of some upland freshwaters.

**Graphical Abstract:**

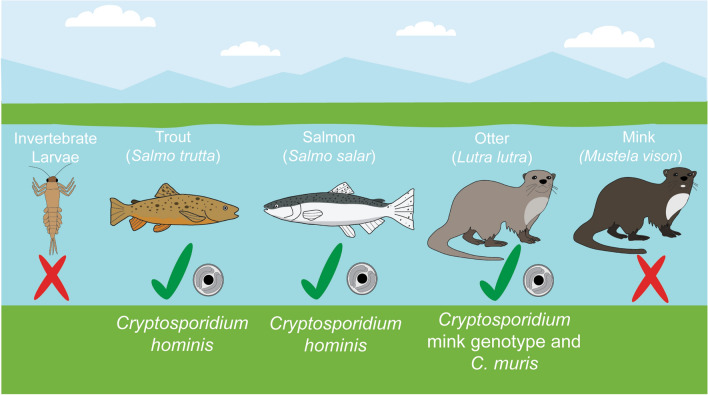

## Background

*Cryptosporidium*, a waterborne protozoan parasite, is an important cause of gastroenteritis globally and the second leading cause of moderate to severe diarrhoea in young children in Southeast Asia and Africa [1]. There is currently no vaccine available to protect against cryptosporidiosis, and therapeutic drugs for the treatment of infection are extremely limited [2]. More than 40 *Cryptosporidium* species have been described, infective to a wide range of hosts, including humans, domestic and wild mammals, birds, reptiles, amphibians and fishes [3]. These parasites transmit between hosts via the faecal-oral route, either directly through contact with an infected individual or indirectly through transmission in faecally contaminated water or, less commonly, food [4, 5]. Water acts as an important medium for the dissemination of *Cryptosporidium* oocysts throughout the environment, and different water resources (including recreational, drinking, groundwater and wastewater) are frequently contaminated with this parasite [6, 7].

*Cryptosporidium* in freshwaters can arise from multiple sources, including runoff from contaminated soil or hard-standings, discharge from wastewater treatment facilities, sewage systems and infected livestock grazing in riparian zones [7, 8]. Young cattle (< 6 weeks) especially contribute to the environmental loading of *Cryptosporidium parvum* while older animals may shed other non-pathogenic *Cryptosporidium* species to humans [9]. During the first days of an active infection, symptomatic calves can excrete 1 × 10^10^ oocysts each day that are infective to other susceptible hosts [10]. This high excretion rate of oocysts favours parasite spread and results in *Cryptosporidium* being ubiquitous in aquatic ecosystems [11]. In upland rural areas, runoff from fields spread with cattle manure as a fertiliser or from fields where cattle are grazing can cause contamination of water courses, but farm management practices, including adequate composting, can reduce the risk [12, 13].

In the event of high rainfall, *Cryptosporidium* oocysts can be mobilised from agricultural land into surface water, including streams and rivers [12]*. Cryptosporidium* oocysts can then remain infective within cool and moist environments for 6 months at temperatures between 0 °C and 20 °C [14, 15]. Once within freshwater ecosystems, the oocysts may stay suspended or settle slowly into river sediments where they have the potential to be resuspended at a later stage [16]. Eukaryotic organisms inhabiting these aquatic environments may act as reservoirs or vectors of *Cryptosporidium* species [6]. Whereas some putative host or vector organisms have been identified, knowledge of the occurrence of *Cryptosporidium* in others is still patchy. For example, *C. parvum* and *C. hominis* oocysts have been isolated from *Acanthamoeba* species and freshwater sponges, respectively [17, 18], while the predation of *Cryptosporidium* oocysts has been demonstrated in free-living ciliated protozoa, amoebae and rotifers [19]. In contrast, little is known about the potential for the aquatic larvae of insects to act as *Cryptosporidium* vectors despite their abundance, diversity and involvement in a wide range of processes in running waters that could bring them into contact with *Cryptosporidium* oocysts.

*Cryptosporidium parvum* has also been detected in the microcrustacean, *Artemia franciscana*, prey of both wild and cultured fish and therefore of potential disease risk to them [20]. Our knowledge of fish-borne cryptosporidiosis is increasing steadily as parasites are being genetically characterised from wild, farmed and ornamental fishes in both marine and freshwater environments ([21], but see [22] for a more detailed review). While is not yet clear whether the presence of these parasite species in fish represents a true infection or mechanical transport, fish could still contribute to the transmission of *Cryptosporidium* through the aquatic food chain [23]. Notably, *Cryptosporidium* oocysts have been reported in the faeces of four species of otter including Eurasian otter (*Lutra lutra*) (in northwest Spain), giant otter (*Pteronura brasiliensis*), neotropical otter (*Lontra longicaudis*) (both in northern Brazil) and the North American river otter (*Lontra canadensis*) (in the Pacific Northwest of the USA). All four otter species are piscivorous predators; whether these parasites can infect and multiply in these host species is unknown [24–26].

This study aimed to evaluate *Cryptosporidium* occurrence and distribution in freshwater hosts from English and Welsh upland river systems. Detecting and characterising human-infecting *Cryptosporidium* species in fish, semi-aquatic mammals and or invertebrate larvae would alert us of the potential pathogen risk in aquatic biota.

## Methods

### UK study area

**Fig. 1:**
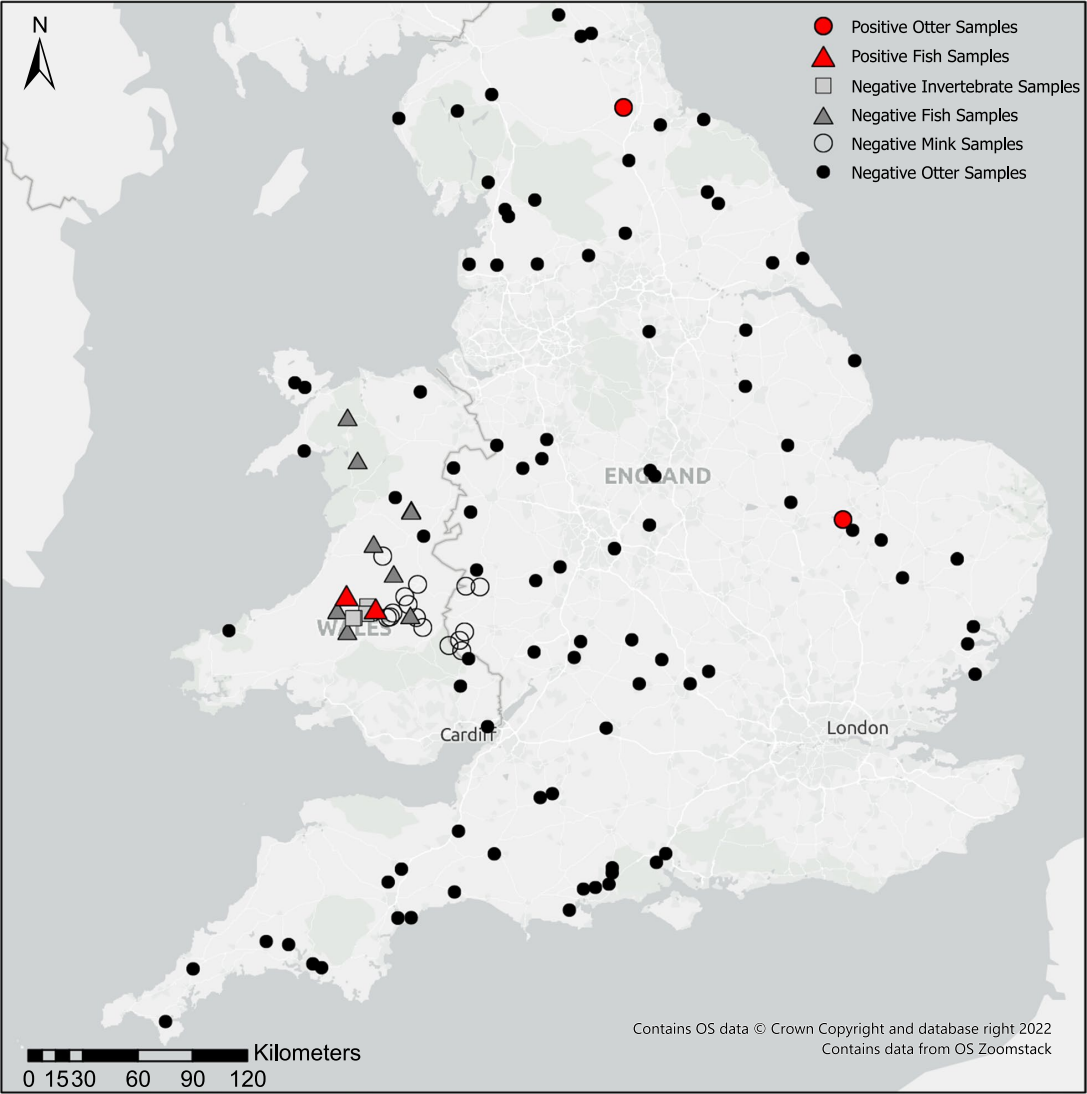
The upland river study sites in England and Wales where fish, semi-aquatic mammals and aquatic invertebrates were collected between 2012 and 2015

The study sites used were drawn partly from the interdisciplinary Natural Environment Research Council (NERC) ‘DURESS’ project ‘Diversity of Upland Rivers for Ecosystem Service Sustainability’ (see Fig. 1 and [27]) and partially from a UK wide surveillance project on otter corpses sampled opportunistically between 2011 and 2015 (Cardiff University Otter Project, https://www.cardiff.ac.uk/otter-project). In combination, the sites encompassed a range of geologies, soil formations, altitudes and land uses, and in many cases are supported by long-term biological and environmental data from stakeholder collaboration with the UK Environmental Agency, Natural Resources Wales, the Welsh Government and UK Forest Research.

### Sample collection

Wild salmon (*Salmo salar,* *n* = 17), trout (*Salmo trutta* *n* = 41) and bullhead (*Cottus gobio*, *n* = 16) were collected under licence from 11 upland river sites across Wales in 2012 by electrofishing. Otter faeces (*n* = 92) were collected from post-mortem examinations (see section UK study area), and mink faecal samples (*n* = 24) were collected during bankside searches. The otter samples comprised faeces from sub-adults and adults. In this study, adult otters are defined as females with signs of reproductive activity or as males with a baculum > 60 mm; sub-adult females are defined as having a body weight > 2.1 kg with no signs of reproductive activity; and sub-adult males are defined as having a body weight > 2.1 kg with a baculum < 60 mm. Aquatic invertebrate larvae (*n* = 390, 109 used for method development and controls, and 281 for testing; Table 1) were collected in 2012 through 2-min-long, semi-quantitative kick sampling of six sites in central Wales, identified to the genus level (9 different genera) and preserved in 100% molecular grade ethanol.

**Table 1:** The occurrence of *Cryptosporidium* species and genotypes detected from animal hosts associated with upland rivers in England and Wales (2012–2015)

Taxa	Sample size	Presence of *Cryptosporidium*			GenBank Accession number
		Positive samples	Prevalence (%)	Species/genotype	
Invertebrate (larvae)					
*Baetis*	16 pools (68 larvae)	0	0	N/A	N/A
*Rhithrogena*	8 pools (35 larvae)				
*Leuctra*	13 pools (52 larvae)				
*Hydropsyche*	3 pools (9 larvae)				
*Simulium*	11 pools (44 larvae)				
*Philapotamus*	2 pools (5 larvae)				
*Plectrocnemia*	4 pools (6 larvae)				
*Amphinemura*	9 pools (41 larvae)				
*Diplectrona*	5 pools (21 larvae)				
Fish (paired gills and guts)					
Salmon (Salmonidae)	17	1 (gut)	5.9	*C. hominis*	OP999667
Brown Trout (Salmonidae)	41	1 (gut)	2.4	*C. hominis*	OP999668
Bullhead (Cottidae)	16	0	0	N/A	
Mammal (faeces)					
Otter (*Lutra lutra)*	92	2	2.1	*C. muris*	OP999670
				*Cryptosporidium* mink genotype	OP999669
Mink (*Mustela vison*)	24	0	0	N/A	

*N/A* Not available

### Evaluation of* Cryptosporidium* detection in invertebrate larvae

To evaluate the sample preparation methods used in the detection of *Cryptosporidium* oocysts from invertebrate larvae, we used an artificial contamination procedure with *C. hominis* oocysts which we predicated would be unlikely to be present naturally in these samples. *Cryptosporidium hominis* oocysts were first purified from anonymised stools using the saturated salt flotation method and enumerated using an improved Neubauer haemocytometer. A sample of field-collected invertebrate larvae was then seeded with oocysts via a Nanoject II injector (Drummond Scientific Broomall, PA, USA) with pulled glass capillary needles into the abdomen (targeted soft tissue between the exoskeleton). In this evaluation, three replicates of the following genera were seeded with 200 oocysts each: the ephemeropterans, *Baetis* and *Rhithrogena*; the trichopteran, *Diplectrona;* the plecopterans, *Amphinemura* and *Leuctra;* and the dipteran, *Simulium*. Three replicates of *Baetis* and *Amphinemura* were also injected with suspensions containing 5, 25, 50 or 100 oocysts. Controls included positive (200 *C. hominis* oocysts with and without the addition of *Baetis* larvae) as well as negative (reverse osmosis water) samples. Prior to DNA extraction, seeded invertebrate larvae were ground up in liquid nitrogen and freeze-thawed in lysis buffer. DNA extractions were performed using the Gentra Puregene Kit according to the manufacturer’s instructions (Qiagen, Hilden, Germany). To detect only the seeded oocysts, we used a real-time *C. hominis*-specific PCR that amplified a 169-bp fragment of the LIB13 locus [34], duplexed with a commercial non-competitive (primer-limited) internal control PCR (PrimerDesign, Southampton, UK). Thermocycling conditions were 95 °C for 10 min, followed by 55 cycles of 95 °C for 15 s and 60 °C for 60 s, using the forward primer 5′-TCCTTGAAATGAATATTTGTGACTCG-3′, the reverse primer 5′-AAATGTGGTAGTTGCGGTTGAAA-3′ and the minor groove binding probe VIC-5′-CTTACTTCGTGGCGGCGT-3′ MGB-NFQ [28].

### Sample preparation, DNA extraction and PCR

Non-seeded invertebrate larvae (281 individual larvae from 9 different genera) were pooled in groups of one to five larvae of the same genus per pool depending on the number collected (Table 1). Each pool was ground in liquid nitrogen and DNA extracted as described above. Paired fish gill samples (*n* = 74) alongside scrapings of the entire intestinal tracts (*n* = 73) were recovered and stored at − 20 °C until processed. DNA was extracted using the QIAamp DNA Mini Kit Tissue protocol as described in the manufacturer’s instructions (Qiagen). DNA was extracted directly from otter and mink faeces using the QIAamp Fast DNA Stool Mini Kit according to the manufacturer’s instructions (Qiagen).

*Cryptosporidium* DNA was identified by nested PCR of the small subunit (SSU) ribosomal RNA (rRNA) gene that detects all *Cryptosporidium* species [29]. A 1325-bp PCR product was amplified first in a primary PCR using the primers 5′-TTCTAGAGCTAATACATGCG-3′ and 5′- CCCATTTCCTTCGAAACAGGA-3′ [36]. PCR conditions involved HotStar Taq (Qiagen) activation at 95 °C for 15 min, followed by 40 cycles of 94 °C for 45 s, 60 °C for 45 s and 72 °C for 1 min, with a final extension at 72 °C for 7 min. In the secondary PCR, a 2-µl aliquot of the primary PCR product (approx. 830 bp) was used as a template for amplification with forward primer 5′-GGAAGGGTTGTATTTATTAGATAAAG-3′ and reverse primer 5′-CTCATAAGGTGCaGAAGGAGTA-3′ [30, 31] under the same cycling conditions as used for the primary PCR except for annealing at 62 °C. The *C. hominis*-specific real-time PCR with a duplexed internal amplification control PCR provided useful information about the efficiency of sample preparation and DNA extraction as well as the potential effect of PCR inhibition from invertebrate larvae. This PCR was not used for screening field samples because it was unknown which *Cryptosporidium* species would be present, so for field samples we used the nested PCR of the SSU rRNA gene, known to provide sensitive and specific amplification of all *Cryptosporidium* species [29]; at the time of the study, a real-time PCR targeting the SSU rRNA gene was not available in our laboratory. *Crptosporidium hominis*-positive samples were also tested by a nested PCR targeting the GP60 locus in an attempt to identify the subtype. The primary PCR was performed using primers 5′-ATAGTCTCCGCTGTATTC-3′ (from [32]) and 5′-GGAAGGAACGATGTATCT-3′ (from [33]). PCR conditions consisted of an initial denaturation at 95 °C for 3 min, 35 cycles of denaturation at 94 °C for 45 s, annealing at 50 °C for 45 s and extension at 72 °C for 60 s, with a final extension of 72 °C for 10 min [34]. A secondary PCR product (800–850 bp) was then amplified from a 2-µl aliquot of the primary PCR product using the primers 5′-TCCGCTGTATTCTCAGCC-3′ and 5′-GCAGAGGAACCAGCATC-3′ (both from [32]), with the same cycling conditions as used for the primary PCR.

DNA sequencing of all SSU rRNA amplicons was performed by Source Bioscience (Cambridge, UK).

### Ethics statement

Fish sample collection was conducted under licence through National Resources Wales (NRW) under Schedule 5 of the Wildlife and Countryside Act (1981, as amended).

## Results

### Evaluation of the detection of* Cryptosporidium* in invertebrate larvae

All three replicate samples of *Diplectrona*, *Simulium* and *Rhithrogena* tested positive for *C. hominis* after being injected with 200 oocysts, while for the other genera examined two of the three replicate samples were positive (Table 2). All *Baetis* and *Amphinemura* larvae seeded with 100 oocysts were positive; for the lower oocyst dosages of 5, 25 and 50 oocysts, at least one replicate sample was negative (Table 3). These results suggested that it was possible to detect oocysts from larvae but that the limit of detection was between 50 and 100 oocysts per extraction, although no oocysts were detected in our unseeded field samples. The cycle threshold (Ct) values between the 200 oocyst *C. hominis* control and the spiked invertebrate larvae matrix were variable, but always higher than the control value (Table 2).

**Table 2:** Molecular detection of *Cryptosporidium hominis* in invertebrate larvae samples seeded with approximately 200 oocysts

Sample	Number of replicates	Number of oocysts seeded	Mean Ct^a^ (± SD)	Number of *Cryptosporidium*-positive samples
*Baetis* sp.	3	200	36.37 (± 1.20)	2
*Diplectrona* sp.	3	200	34.94 (± 1.38)	3
*Simulium* sp.	3	200	37.88 (± 0.52)	3
*Amphinemura* sp.	3	200	38.45 (± 0.48)	2
*Leuctra* sp.	3	200	37.05 (± 0.06)	2
*Rhithrogena* sp.	3	200	36.68 (± 1.77)	3
*Baetis* sp. Control	1	200	35.12	1
Negative control	1	0	Negative	0
*C. hominis* Control	1	200	33.36	1

* Ct *Threshold cycle (number of cycles required to detect fluorescence of a sample in real-time quantitative PCR,* SD* standard deviation

**Table 3:** Molecular detection of *Cryptosporidium hominis* in invertebrate larvae samples seeded with approximately 5, 25, 50 or 100 oocysts

Sample	Number of replicates	Number of oocysts seeded	Mean Ct (± SD)	Number of *Cryptosporidium*-positive samples
*Baetis* sp.	1	100	36.60 (± 1.73)	3
*Baetis* sp.	1	50	36.24 (± 1.86)	3
*Baetis* sp.	1	25	Negative	0
*Baetis* sp.	1	5	38.44 (± 0.18)	1
*Amphinemura* sp.	1	100	35.28 (± 0.18)	3
*Amphinemura* sp.	1	50	37.98 (± 1.85)	1
*Amphinemura* sp.	1	25	37.56	1
*Amphinemura* sp.	1	5	37.4	1
Negative control	1	0	Negative	0
*C. hominis* Control	1	200	32.92	1

* Ct *Threshold cycle (number of cycles required to detect fluorescence of a sample in real-time quantitative PCR,* SD* standard deviation

### Occurrence of *Cryptosporidium* species in riverine hosts

*Cryptosporidium* species were detected in four out of 471 (0.8%) samples from freshwater biota collected from the full array of English and Welsh sites. The parasites were found in 2/74 fish and 2/116 mammals sampled, but not in the nine genera of aquatic invertebrates (Table 1). Screening of fish gut samples identified *C. hominis* in one salmon and one trout individual, by SSU rRNA gene sequencing. Unfortunately, these *C. hominis* samples failed to amplify when tested by the LIB13 and GP60 assays. Of the 92 otter faecal specimens tested, two samples were positive, one for *C. muris* and the other for *Cryptosporidium* mink genotype. *Cryptosporidium* was not detected in the 24 mink samples.

## Discussion

This study investigated the occurrence of *Cryptosporidium* spp. in some of the fauna inhabiting rivers within England and Wales. We detected *Cryptosporidium* species, including one pathogenic to humans, in Atlantic salmon (*C. hominis*), brown trout (*C. hominis*) and otters (*C. muris* and *Cryptosporidium* species mink genotype). Although *Cryptosporidium* was not detected in the nine genera of aquatic invertebrates sampled, we demonstrate that molecular screening for *Cryptosporidium* in aquatic invertebrate larvae is achievable, although the limit of detection does need to be improved. Such invertebrates may still serve as mechanical vectors for disseminating oocysts, as has been shown for wild filth flies [35], but whether *Cryptosporidium* naturally occurs in invertebrates remains unknown. In a single study in northwestern Spain, samples containing a community of aquatic invertebrate nymphs and larvae were ground, filtered and screened by immunofluorescent microscopy for *Cryptosporidium* [36]. The authors of the study detected oocysts in four of these samples, but the number of oocysts detected was not reported and so could be below the limit of detection of our assay. Furthermore, the invertebrate host could not be identified as the whole community was tested together [36].

In the present study, *C. hominis* oocysts were found in both trout and salmon, confirming the presence of this protozoan parasite in English and Welsh rivers. The lack of amplification at the LIB13 and GP60 loci may well be due to the sensitivity of the assay if low numbers of parasites were present, but we cannot exclude the presence of a *C. hominis* variant that does not amplify with those more specific primer sets. This human pathogenic *Cryptosporidium* species is considered to be anthroponotic, and previous reports in animals are rare, suggesting water contamination arose from wastewater [37]. This is not unheard of in upland waters; an outbreak of *C. hominis* in northwestern Wales was directly linked to inadequate wastewater treatment [38]. Given the rural nature of the study sites, it is likely that *C. parvum* was present in the river catchments, but the levels would be influenced by the number, type and age of livestock and their husbandry, the time of year, weather events and geographical features of the catchment as well as wastewater inputs.

Limited data are available on the taxonomy, epidemiology and distribution of *Cryptosporidium* species and genotypes in fish [39]. Highly variable parasite prevalences have been reported, ranging between 0.8% and 100%, with the top end of this range mainly found in farmed juveniles, while larger fish generally show fewer infections over time [40]. Human *Cryptosporidium* species have been detected at consistently low prevalences (< 1%) in fish hosts [6]. However, to fully assess the prevalence of *Cryptosporidium* species in fish, further sampling from areas with high levels of human or agricultural faecal contamination is required. There have been inconsistent reports as to whether fish act as natural hosts for human infecting *Cryptosporidium* species [40–42]. Although there are no known reports of fishborne cryptosporidiosis in humans at present, *C. parvum* oocysts were detected from commercial Atlantic blue crabs (*Callinectes sapidus*) during animal handling and preparation [43]. Foodborne cryptosporidiosis following the ingestion of these crabs will most likely be prevented during cooking processes, but nevertheless parasite exposure can still result in the contamination of storage areas potentially leading to future disease. Furthermore, 56% of hand swabs taken from urban anglers in Baltimore were positive for *Cryptosporidium* species [44]. Thus, handling of *Cryptosporidium*-contaminated animals, including fish, may pose a significant infection risk to humans.

Within freshwater and marine foraging otter populations, reported *Cryptosporidium* prevalence ranges from 3.9% to 41.7% [24–26]; our findings are at the lower end of this range, at 2.17%, but the spatial distribution of the *Cryptosporidium*-positive otter samples suggests that *Cryptosporidium* could be widespread. Although *Cryptosporidium* oocysts have been detected in these aquatic mustelids, the parasite species are not routinely identified. This study is the first report of the *Cryptosporidium* sp. mink genotype and *C. muris* in otters. The habitat and diets of the introduced American mink and Eurasian otter overlap to some extent [45], and both species prey on small rodents known to harbour *C. muris* (although consumption of rodents by otters is relatively rare; see [46]). Interestingly, the *C. muris*-positive otter did have a rat’s tail in its stomach upon post-mortem examination. Parasite transmission may therefore occur throughout the food chain. Alternatively, otters may have been exposed to both parasite species following the ingestion of faecally contaminated water. As with fish, the presence of these parasites in Eurasian otters does not necessarily indicate an active infection, and it remains unknown whether otters are true hosts of these *Cryptosporidium* species or merely another reservoir of infection.

The current study has several limitations. First, as detection of *Cryptosporidium* species was based solely upon PCR assays and, therefore, the detection of *Cryptosporidium* DNA, there is no direct evidence that the positive fish and otter samples contained the parasite transmission stage, the oocyst. Secondly, as there was no quantification of the positive samples, we do not know whether these hosts had a low or high burden of *Cryptosporidium*, or, quite possibly in the case of the *C. hominis*-positive fish, whether the parasite was passing through the gut rather than arising from an active infection. Thirdly, as sampling relied on collection from two separate projects covering different study sites, the spatial distribution of the positive samples could not be analysed. Fourthly, the oocyst limit of detection in invertebrate larvae was relatively high, quite feasibly more than would realistically be expected to be ingested by the larvae. The limit of detection might be influenced by the invertebrate sample matrix through PCR inhibition or losses during DNA extraction, which could explain the variability between Ct values of the different insect genera and the positive control.

## Conclusions

We detected *Cryptosporidium* species in 0.8% of samples from freshwater biota tested in this study. More specifically, the human-infective species, *C. hominis*, was detected in fish as well as *C. muris* and *Cryptosporidium* species mink genotype in otters. The low detection rate of *Cryptosporidium* species known to be pathogenic to humans may indicate that, in the areas sampled, there is a low risk of human infection from freshwater biota. Further investigation is needed to determine whether the occurrence of *Cryptosporidium* species and genotypes found in this study represent true infections or whether these animals act as transport vectors, and to further refine detection of oocysts in the plethora of potential invertebrate hosts.

## Data Availability

Data is publicly available at the UK CEH Environmental Information Data Centre and can be accessed via the following: 10.5285/84242834-dc78-49a6-83cb-951edac65d18. Sequence data presented in this study, deposited in the NCBI GenBank database, is openly available under Accession numbers: OP999667, OP999668, OP999670 and OP999669.
