# Correction: The occurrence and zoonotic potential of *Cryptosporidium* species in freshwater biota

**DOI:** 10.1186/s13071-023-05987-8

**Published:** 2023-10-19

**Authors:** Laura Hayes, Guy Robinson, Rachel M. Chalmers, Steve J. Ormerod, Anna Paziewska-Harris, Elizabeth A. Chadwick, Isabelle Durance, Jo Cable

**Affiliations:** 1 School of Biosciences, Cardiff University, Cardiff, CF10 3AX UK; 2 Cryptosporidium Reference Unit, Public Health Wales Microbiology, Singleton Hospital, Swansea, SA2 8QA UK; 3 Swansea Medical School, Swansea University, Swansea, SA2 8QA UK; 4 Lukasiewicz Research Network, PORT Polish Centre for Technology Development, Stablowicka 147, 54-066 Wroclaw, Poland

**Correction: Parasites & Vectors (2023) 16:209** 10.1186/s13071-023-05827-9

Following publication of the original article [1], the author flagged that ‘*Mustela lutreola*’ had been erroneously used instead of ‘*Mustela vison*’ in Table 1 and the graphical abstract of the article. The article has since been updated to correct this. The authors thank you for reading and apologize for any inconvenience caused.
